# Remarkable Exemption of Buffalo from Epidemic Cholera and the Usual Diseases of the Season

**Published:** 1866-11

**Authors:** 


### Editorial Department.

### Remarkable Exemption of Buffalo from Epidemic Cholera and the usual diseases of the season.

It is quite remarkable that cholera should prevail to so great extent in almost all the larger cities of the country and so few cases of choleraic disease be reported in Buffalo. Three or four cases thus far comprise the whole, and these perhaps hardly come within the present fashionable definition of this disease. The new work on Cholera by John C. Peters, who gives his own opinions in twelve distinct expressions, which are maintained, as he says, by the most scientific and experienced physicians of the times, will exclude most or all of our cases of cholera, though there can be no doubt that the general symptoms of cholera were present.

Article 6th says: No amount of filth, imprudence or diarrhœal disease without the addition of this peculiar cause, (choleraic poison) will give rise to true Asiatic cholera in temperate climates. 12th. Finally, no case of diarrhœa, cholera morbus or dysentery can be converted into cholera, unless the patient has also been exposed to the peculiar infection of this disease.

The cases in Buffalo, reported as cholera, were not supposed to have had any exposure to the "peculiar infection" of cholera, that is, the most perfectly marked and unquestionable ones were known not to have been exposed to the infection of cholera. One patient we believe was supposed to have contracted the disease in New York, which only completed its course in Buffalo; perhaps one other also contracted the disease in another city and came here to die.— It was under the care of an irregular physician, and of its character or symptoms we have no knowledge; the daily papers announced death from "typhoid fever, following cholera." The absurdity of this, reminds us of a good story told by Rev. Dr. H—— of Buffalo, who is not as much given to being deceived in matters of medicine, as most men who have his titles, and we may be pardoned for digressing enough to repeat the Doctor's experience. One morning during the prevalence of one of the former epidemics of cholera, meeting a newly converted homœpathic parishioner, who was very ardent for the newly discovered wonders of the infinitesimal, and anxious to make a startling announcement, inquires, "have you heard of the wonderful cure Dr. Warner has made?" On being told that he had not, he says, "Dr. Warner has cured a man of cholera after he had been in collapse twelve hours." Dr. H. replied reverently, that he did not believe any one but God himself could do such a thing. But it has been done, and when all hope of his recovery was given up, was the positive reply. Who was the fortunate object of this miraculous cure? inquires the Doctor. It was er, it was er, Mr. —— on Pearl street. My dear Sir, I attended that man's funeral myself, two weeks or more since. Ye—s, he's dead, I know, but Dr. Warner cured him of the cholera, and then he died from the effects of the medicine the old school doctors had given him before Dr. Warner was called. The doctor's wholesome advice to his quite prominent parishioner may be imagined, while we return to the question of what shall, and what shall not, be regarded as cholera. One of the most well marked cases, under the care of as careful and intelligent observation as the country affords, without any exposure to the infection of cholera, and in as healthy a location as is to be found in the city, terminates fatally, and is pronounced "cholera, and nothing else but cholera," by a most discriminating and experienced physician. The inference is plain, that either this, and other similar cases, cannot properly be called cholera, or the present prevailing code concerning this disease requires modification. That the patient had the usual symptoms of the disease, in severe and fatal form, cannot be doubted; but that it lacked the exposure to infection from choleraic disease is also certain, and therefore according to the present view of all those who believe contagion necessary for true cholera could not come under that name. The question of whether we shall call such disease cholera is a proper one for consideration, and the arguments for and against may very properly be stated. The sudden appearance and disappearance of cholera here and there, following laws thus far, as we believe inscrutable, induces us to withhold assent to all theories of its communication, hoping at length to arrive at conclusions in perfect harmony with facts.

We have enjoyed a season of the most remarkable exemption from all forms of disease, fewer cases of cholera morbus, diarrhœa and dysentery having occurred in Buffalo than for many years past. The announcement of cholera is generally received with distrust, the demand that true cholera be epidemic, being general. If cholera lacks the epidemic character, it does not quite answer public expectation, and perhaps it might be as well to add the *morbus* to it, and let it pass, unless it does show disposition to extend itself, whatever other symptoms it presents. Others think differently—that it is better to call such sporadic cases, cholera, that the community may be aware of the presence of the disease and act accordingly.

